# Is cancer progression caused by gradual or simultaneous acquisitions of new chromosomes?

**DOI:** 10.1186/s13039-017-0350-4

**Published:** 2018-01-15

**Authors:** Mathew Bloomfield, Peter Duesberg

**Affiliations:** 1 Department of Molecular and Cell Biology, Donner Laboratory, University of California at Berkeley, Berkeley, CA 94720 USA; 2 Present address: Department of Natural Sciences and Mathematics, Dominican University of California, San Rafael, CA USA

**Keywords:** Saltational progression, Metastasis, Cancer drug-resistance, Cell fusion, Hybridoma, Aneuploidy-catalyzed karyotype variation

## Abstract

**Background:**

Foulds defined, “Tumor progression (as a) permanent, irreversible qualitative change in one or more of its characters” (Cancer Res. 1954). Accordingly progressions, such as metastases and acquired drug-resistance, were since found to be subspecies of cancers with conserved and numerous new chromosomes. Here we ask whether cancers acquire numerous new chromosomes gradually or simultaneously in progressions. The currently prevailing theory of Nowell (Science, 1976) holds that unexplained “genetic instability” generates “variant sublines (with) changes in chromosome number” and that “clonal” progressions arise by “stepwise selection of more aggressive sublines”. The literature, however, contains many examples of “immediate” selections of progressions with numerous new chromosomes - notably experimentally initiated fusions between cancers and heterologous cells. Furthermore, the stepwise progression theory predicts intermediate sublines of cancers with multiple non-clonal additions of new chromosomes. However, the literature does not describe such intermediates.

**Results:**

In view of these inconsistencies with stepwise progression we test here a saltational theory, in which the inherent variability of cancer-specific aneuploidy generates “immediate” progressions with individual clonal karyotypes, transcriptomes and phenotypes in single steps. Using cell fusion as an established controllable model of “immediate” progression, we generated seven immortal murine hybridomas by fusing immortal murine myeloma cells and normal antibody-producing B-cells with polyethylene glycol within a few minutes. These immortal hybridomas contained individual sets of 71 to 105 clonal chromosomes, compared to the 52 chromosomes of the parental myeloma. Thus the myeloma had gained 19 to 53 new clonal chromosomes in seven individual hybridomas in a single step. Furthermore, no stable intermediates were found, as would be predicted by a saltational process.

**Conclusions:**

We conclude that random fusions between myelomas and normal B-cells generate clonal hybridomas with multiple, individual chromosomes in single steps. Similar single-step mechanisms may also generate the “late” clonal progressions of cancers with gains of numerous new chromosomes and thus explain the absence of intermediates. Latency would reflect the low probability of rare stochastic progressions. In conclusion, the karyotypic clonality of hybridomas and spontaneous progressions suggests karyotypic alterations as proximate causes of neoplastic progressions. Since cancer-specific aneuploidy catalyzes karyotypic variation, the degree of aneuploidy predicts the clinical risk of neoplastic progression*, c*onfirming classical predictions based on DNA content**.**

## Background

Foulds defined, “Tumor progression (as a) permanent, irreversible qualitative change in one or more of its characters” [1]. Accordingly several labs including ours have recently shown that progressions such as metastases and drug-resistant variants are actually clonal subspecies of cancers with parental and typically numerous new chromosomes [2–13].

Here we ask whether multiple new chromosomes of progressions are acquired gradually or simultaneously in one-off events.

The currently prevailing theory of Nowell (Science, 1976) holds that unexplained “genetic instability” generates “variant sublines (with) changes in chromosome number” and that “clonal” progressions arise by “stepwise selection of more aggressive sublines” [14]. The literature, however, contains numerous examples of selections of “immediate” progressions [14] with multiple new chromosomes [7, 15–18] - notably experimentally initiated fusions between cancers and heterologous cells [18–26]. Furthermore, the prevailing stepwise theory predicts stable intermediate sublines of cancers with multiple non-clonal additions of new chromosomes. However, the literature does not support the existence of non-clonal intermediates [14, 26, 27].

### Alternative single-step theory of progression

In view of these inconsistencies with stepwise progression we test here a single-step or saltational theory of progression, in which the inherent instability of cancer-specific aneuploidy catalyzes steady karyotypic variations in single steps automatically by unbalancing thousands of balance-sensitive genes. Most of these variants alter parental cancer karyotypes within clonal margins of cancer-specific autonomy, typically by the gain or loss of single copies of chromosomes, while others lose autonomy and thus perish [9, 28–31].

A small minority of these random karyotypic variations would however, acquire new autonomous clonal karyotypes, transcriptomes and phenotypes, which are still related to, but distinct from parental predecessors [9–13, 32]. These new subspecies or progressions are also clonally stabilized by selections for cancer-specific autonomy, just like parental cancers are [9–12, 28, 29, 33, 34].

Using cell fusion as an established controllable model of “immediate” progression, we generated seven individual murine hybridomas of immortal murine myeloma cells and normal antibody-producing B-cells by fusing these cells with polyethylene glycol in a virtually immediate fusion process of minutes [21, 23, 25, 35]. Such progressions would thus be new clonal subspecies of parental cancers.

A saltational mechanism of progression would make three testable predictions: (1) Time-independent progressions with unpredictable numbers of chromosomes at low stochastic rates – just like de novo carcinogenesis [9]. (2) As per definition the saltational mechanism would also predict the absence of stable intermediates [9, 11, 30]. (3) The theory would also predict spontaneous progressions of progressions on the same principles as primary progressions.

**Fig. 1:**
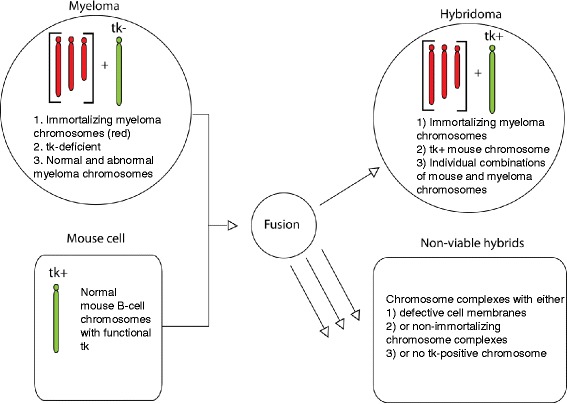
Generation of mouse hybridomas by fusions of immortal thymidine kinase(tk)-less mouse myeloma cells with normal mouse B-cells in about 50% polyethylene glycol [26, 35, 36]. After fusions of 5 to 10 min immortal myeloma-B-cell hybrids or hybridomas survive in the presence of the inhibitor of DNA synthesis aminopterin on supplemental thymidine picked up by B-cell-derived kinase. The majority of fused cells die from defective cell walls randomly denatured by polyethylene glycol or from non-proliferative chromosome combinations without myeloma-specific immortalizing chromosomes and or from lack of chromosomes with B-cell specific kinase

In an effort to distinguish between a single step and multi-step theories, we tested an established experimental system of “immediate” progression [14], namely the immortalization of antibody-producing murine B-cells by fusion (or cell hybridization) with immortal murine myeloma cells to “hybridomas” [21, 23, 35] (Fig. 1). In this system, fusions of immortal myeloma cells convert normal B-cells to immortal clonal hybridomas in a few minutes in the presence of inactivated Sendai virus or polyethylene glycol- at rates of 10^-4 to 10^-5 hybridoma per myelolma cells [23, 35–37]. This short reaction time effectively limits fusion events to a single step process [21, 23, 35]. The resulting hybridoma clones are indeed already known to have new hybrid karyotypes [23, 26, 38] (Fig. 1). To test the predictions of our theory that simultaneous acquisitions of multiple new chromosomes may generate clonal progressions or subspecies in single steps, we prepared and analyzed the chromosomes and phenotypes of seven new immortal hybridomas.

In short we found that all seven hybridomas were individual subspecies of the parental myeloma with numerous new clonal chromosomes and that there were no karyotypic intermediates. These results support a saltational process of cancer progression.

## Results

In the following we describe: (a) The preparation of seven hybridomas as models of immediate saltational progressions by experimental fusions of immortal murine myeloma Ag8 cells and normal B-cells (Fig. 1 and Methods). (b) Evidence for individual phenotypes of these hybridomas, which the saltational theory postulates based on selection of random recombinations of chromosomes of two or more cells hybridized by fusion. (c) Evidence for the clonality and individuality of the karyotypes of hybridomas, which the saltational theory postulates based on the low probability that random fusions of chromosomes of two types of cells generate a new immortal hybridoma species.

### Preparation of hybridomas

Our colleagues Jennifer Zeitler and Robert Beatty kindly offered to us seven hybridomas from their undergraduate course in immunology here at UC Berkeley. Following published procedures, these hybridomas were prepared by fusions of immortal mouse myeloma Ag8 cells without functional thymidine kinase genes with equal amounts of normal thymidine kinase-positive B-cells and selections for immortal thymidine-dependent hybridoma clones in the presence of aminopterin, an inhibitor of de novo thymidine synthesis [21, 23, 26, 35, 36] (Fig. 1, Methods). Based on these procedures our myeloma and B-cells were fused with polyethylene glycol for several minutes, then washed and incubated in selective medium containing aminopterin and thymidine. As shown graphically in Fig. 1, under these conditions only cell hybrids between myeloma-specific immortalizing chromosomes (defined below) and B-cell-derived thymidine kinase-positive chromosomes survive. By contrast un-fused myeloma cells perish, because de novo DNA synthesis is inhibited by aminopterin or because cells are damaged by polyethylene glycol [35, 38]. At the same time un-fused B-cells perish spontaneously in cell culture in a few cell generations.

As described previously, only about one in 10^4 – 5 myeloma Ag8 cells is converted to an immortal hybridoma cell by fusion with equal amounts of B-cells under these conditions [23, 35–37]. These low yields of progressions or subspeciation from myeloma to hybridoma are consistent with the low probabilities to generate new autonomous subspecies by random variation of the chromosomes of an existing species [9–11, 28, 33, 34] (Background).

Within one to two weeks after fusion we first detected hybridoma clones emerging in this selective medium as microscopic clones. Seven of such hybridoma clones were then grown to about 10^6 cells for karyotypic and phenotypic analyzes, typically about a month after fusion or later [23, 26, 36].

As shown in Table 1, three of these seven hybridomas were confirmed to produce antibodies against the specific antigens used to immunize the mice from which the B-cells derived by our colleagues Zeitler and Beatty, and hence termed Hyb CN-13 ab+, Hyb cl-12 ab + and Hyb cl-9 ab+. Table 1 also lists the remaining four hybridomas that were not tested for the production of antibodies against inducing antigens and thus labeled Hyb H12 ab-, Hyb F3 ab-, and Hyb 94 and Hyb 1-5 for reasons described below.

**Table 1:** Average clonal chromosome numbers of the mouse, of murine myeloma Ag8 and of seven myeloma-mouse B-cell hybridomas

Myeloma and Hybridoma clones	Clonal chromosome number ± SD	Gains of chromosomes compared to the 52 of myeloma	Gains / losses of chromosomes compared to the 92 of a theoretical myeloma-B-cell hybrid
Mouse	40	–	–
Myeloma Ag8	52 ± 1	–	–
Hyb CN-13 ab+	85 ± 2	33	- 7
Hyb cl-12 ab+	86 ± 9	34	- 6
Hyb cl-9 ab+	105 ± 11	53	+ 13
Hyb H12 ab-	71 ± 2.5	19	- 21
Hyb F3 ab-	79 ± 2	27	- 13
Hyb 94	74 ± 5	22	- 18
Hyb 1-5	99 ± 7.5	47	+ 7

### Clonal phenotypes of hybridomas

**Fig. 2:**
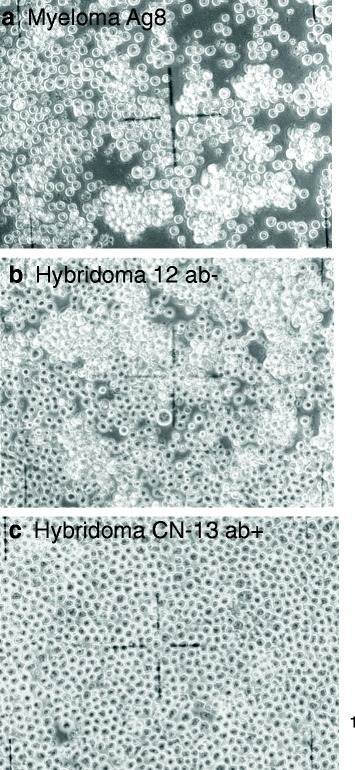
**a, b, c** Cell morphology of murine myeloma Ag8 (**a**), hybridoma Hyb H12 ab- (**b**) and hybridoma Hyb CN-13 ab + (**c**) with phase contrast microscopy at 200× magnification in cell culture. The cells are growing in suspension in medium RPMI 1640 (Methods). Under these conditions Ag8 myeloma and hybridoma Hyb H12 ab- cells form clumps of loosely attached cells, while all hybridoma Hyb CN-13 ab + are settled on the bottom of the culture dish

To test our theory that hybridomas are individual, clonal subspecies of myelomas with individual phenotypes [10, 11], we first looked at cell morphologies. As shown in Fig. 2a-c the cells of the myeloma Ag8 and of the two hybridomas Hyb H12 ab- and Hyb CN-13 ab + were spherical, like all other hybridomas (not shown) and thus hard to distinguish from each other morphologically - in contrast to the distinct 2-dimensional morphologies of cells from solid cancers attached to culture dishes as described by us elsewhere [11]. Nevertheless, both myeloma Ag8 and hybridoma Hyb H12 ab- differed from Hyb CN-13 ab + in forming 3-dimensional aggregates of cells in suspension, in which they are attached to each other. The non-attached cells settled at the bottom of the dish. In contrast all Hyb CN-13 ab + cells formed a dense layer of loose cells at the bottom of the dish. In addition Hyb CN-13 ab + cells were on average a bit larger than Hyb H12 ab- and myeloma cells.

Furthermore, Table 1 shows that some hybridomas differed from others in the production of specific antibodies, e.g. Hyb CN-13 ab+, Hyb cl-12 ab+, Hyb cl-9 ab+. By contrast, Hyb H12 ab- and Hyb F3 ab- are probably antibody-negative, although they were not directly tested, for two reasons: 1) As shown below in Fig. 6, they both lacked intact copies of murine chromosome 12, which encodes the heavy chain of mouse antibodies [39], and 2) The parental myeloma Ag8 of the hybridomas studied here also lacks functional antibody genes [40]. It would follow that both of these clones are antibody-negative.

Moreover the seven hybridomas could be distinguished by individual growth rates (data not shown). For example, hybridomas Hyb H12 ab-, Hyb F3 ab- and Hyb 94 grew about twice as fast as the three anti-body-producing hybridomas Hyb CN-13 ab+, Hyb cl-12 ab+, Hyb cl-9 ab + and the hybridoma Hyb 1-5 (Table 1). These individualities of our hybridomas confirmed and extended earlier observations by Kohler and Milstein [23].

In sum, we conclude that the seven hybridomas have descriptively and functionally distinct clonal phenotypes.

Next we set out to determine whether the chromosomes of our hybridomas were indeed individual and clonal as predicted by the saltational theory.

### Are the chromosomes of hybridomas individual and clonal as predicted by the saltational theory?

The saltational theory of the origin of progressions predicts that each progression of a clonal cancer is a new, individual sub-clone with clonal parental and new progression-specific chromosomes. To test this prediction of the saltational theory of progression, we asked whether the seven hybridomas each contained individual sets of clonal chromosomes.

To answer this question chromosome numbers of individual hybridoma cells were determined from karyotypes prepared from metaphase chromosomes. Owing to the inherent clonal heterogeneity of the chromosome numbers of cancer karyotypes, generated by cancer-specific aneuploidy (see Background, Alternative single-step theory of progression), we used averages of the primary chromosome numbers of five individual cells as standards of clonality.

**Fig. 3:**
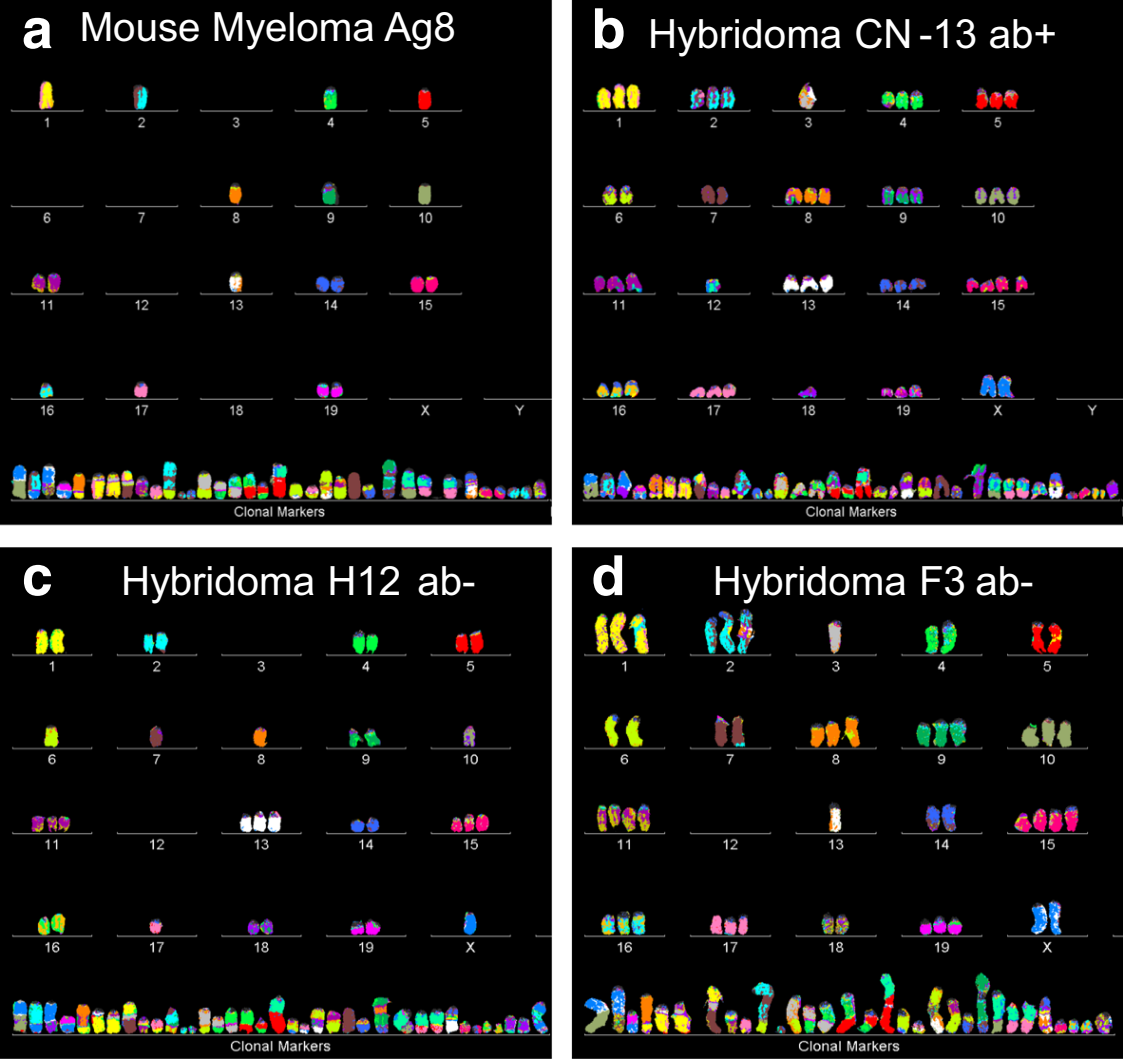
**a**, **b**, **c**, **d** Karyotypes of murine myeloma Ag8 (**a**), and three hybridoma subspecies of myeloma Ag8, Hyb CN-13 ab + (**b**), Hyb H12 ab- (**c**) and Hyb F ab- (**d**). It can be seen that all four immortal clones shared the myeloma-specific set of about 31 marker chromosomes, which define the karyotype of the immortal myeloma clone, from which the hybridomas were derived

Examples of individual karyotypes of three hybridomas, namely hybridomas Hyb CN-13 ab+, Hyb H12 ab- and Hyb F3 ab-, and of the parental myeloma Ag8 are shown in Fig. 3a-d. As can be seen in this figure, each immortal hybridoma contained individual chromosome numbers, as predicted by the theory that hybridomas are individual subspecies of the myeloma. Moreover the individual numbers of chromosomes of these karyotypes already indicated that each hybridoma apparently contained considerably more chromosomes than the parental myeloma, although clonality had yet to be determined.

To determine clonality the chromosome numbers, five individual cells of each hybridoma and parental myeloma were compared in 3-dimensional tables, termed ‘karyotype arrays’ [11]. Such arrays list the numbers of all intact and marker chromosomes on the x-axis, the copy numbers of the chromosomes on the y-axis, and the numbers of karyotypes (K) analyzed on the z-axis. The resulting 3-dimensional arrays show clonality as parallel lines, which are formed by chromosomes from distinct cells with the same copy numbers. At the same time, non-clonal chromosomes show up as readily detectable non-parallel lines in karyotype arrays.

**Fig. 4:**
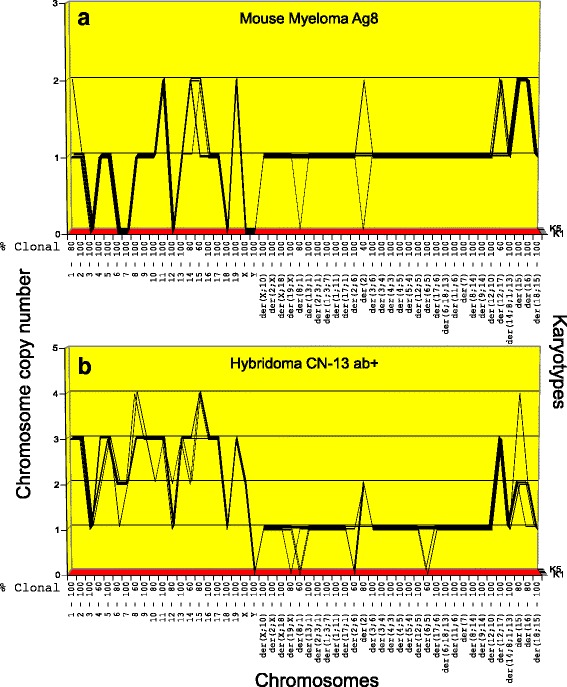
**a**, **b** Karyotype-arrays of mouse myeloma Ag8 (**a**) and the corresponding hybridoma subspecies Hyb CN-13 ab + (**b**). Karyotype-arrays compare the copy numbers of individual chromosomes of multiple karyotypes of a potential cell clone in three-dimensional tables. The tables list the chromosome numbers of arrayed karyotypes, K1 to K5, on the x-axis, the copy numbers of each chromosome on the y-axis, and the number of the five karyotypes arrayed on the z-axis, as described by us [9, 11] and others [12]. Since chromosomes with the same copy numbers form parallel lines in 3-dimensonal karyotype arrays they visually identify clonality. The clonality of each chromosome in percent is listed on the abscissa of each array. Here we compared the karyotype array of myeloma Ag8 (**a**) to that of an antibody-producing (ab+) hybridoma subspecies Hyb CN-13 ab + (**b**). As can be seen in Fig. 4 and Table 2, hybridoma Hyb CN-13 ab + shared with the parental myeloma about 31 highly clonal, myeloma-specific marker chromosomes. In addition the hybridoma shared with the parental myeloma clonal copies of all myeloma-specific normal mouse chromosomes, although the copy numbers of shared mouse chromosomes were 2-3-fold higher in the hybridoma than in the myeloma. By contrast the myeloma lacked several normal mouse chromosomes. Based on the shared clonal myeloma-specific marker and normal mouse chromosomes, the hybridoma Hyb CN-13 ab + is a subspecies of the myeloma and the murine B-cell. It is consistent with the complete set of normal mouse B-cell chromosomes of this hybridoma that it produced antibodies

**Fig. 5:**
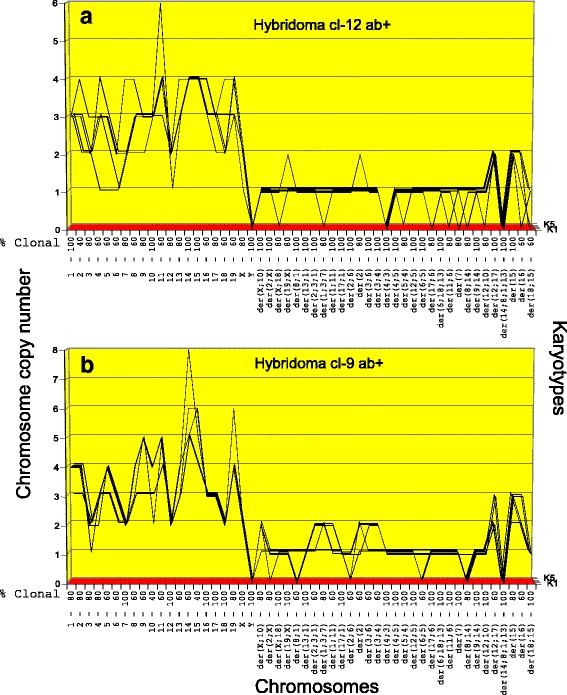
**a**, **b** Karyotype-arrays of five cells (K1 to K5) of hybridoma Hyb cl-12 ab + (**a**) and hybridoma Hyb cl-9 ab + (**b**). As described in Fig. 4 karyotype-arrays reveal the clonality of cancer-specific chromosomes based on the percentage of cells with chromosomes that form parallel lines and thus have identical copy numbers. The arrays of hybridoma Hyb CN-13 ab + and of hybridoma Hyb cl-9 ab + shared highly clonal copies of all 31 myeloma-specific, abnormal marker chromosomes described in Fig. 4a and Table 2. They also share highly clonal copies of all normal mouse chromosomes from the parental B-cell, although at individually distinct copy numbers. Based on the shared clonal myeloma-specific and clonal normal mouse chromosomes shown in Table 2, the two hybridomas are individually distinct clonal subspecies of the myeloma and normal B-cell. The presence of complete sets of normal mouse chromosomes in both hybridomas is consistent with their production of mouse anti-bodies

**Fig. 6:**
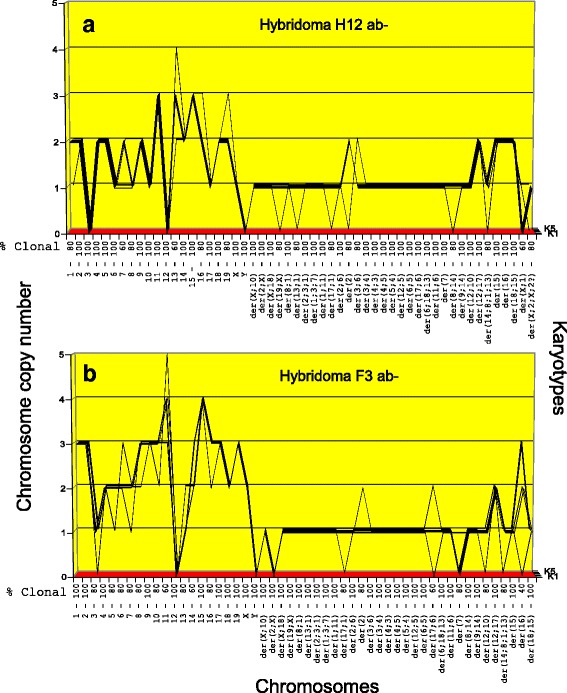
**a**, **b** Karyotype-arrays of five cells of hybridoma Hyb 12 ab- (**a**) and hybridoma Hyb F3 ab- (**b**). The arrays of hybridoma Hyb 12 ab- and of hybridoma Hyb F3 ab- shared highly clonal copies of the 31 myeloma-specific marker chromosomes described in Fig. 4. They also shared highly clonal copies of all normal mouse chromosomes from the parental B-cell, although at individually distinct copy numbers. Based on the shared clonal myeloma-specific and normal mouse chromosomes (see Table 3), the two hybridomas are individually distinct, clonal subspecies of the myeloma and normal B-cells. The absence of normal mouse chromosome 12, which encodes the heavy chain of mouse antibodies in both hybridomas explains their failure to produce of mouse antibodies (see text, Are the chromosomes of hybridomas individual and clonal as predicted by the saltational theory?)

**Fig. 7:**
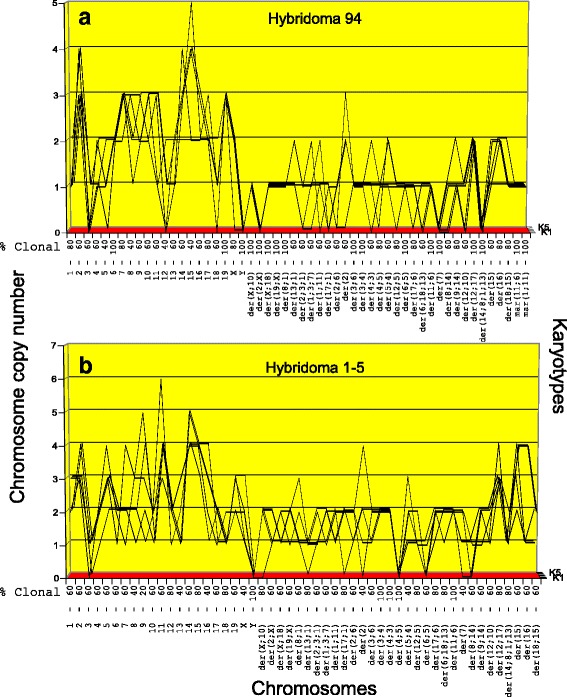
**a**, **b** Karyotype-arrays of five cells of hybridoma Hyb 94 (**a**) and hybridoma Hyb 1-5 (**b**). Both hybridomas are clonally heterogeneous with chromosomal clonalities ranging from 40 to 100%. For example, the individual chromosome numbers of the five hybridoma Hyb 94 cells analyzed range from 71 to 82 for a clonal average of 74 (Tables 1 and 3). Nevertheless, all five Hyb 94 karyotypes shared 28 of the 31 myeloma-specific marker chromosomes and two Hyb 94-specific marker chromosomes (Table 3). The karyotype array of Hyb 1-5 was also relatively heterogeneous. Nevertheless, all five Hyb 1-5 karyotypes shared 30 of the 31 myeloma-specific marker chromosomes with the parental myeloma. In addition they shared all normal murine chromosomes with the parental mouse B-cell and some also with the parental myeloma. The simplest explanation for the high clonal heterogeneity of Hyb 94 and Hyb 105 suggests that these clones are still evolving precursor clones that are losing non-stabilizing chromosomes

In the following we show the karyotype arrays of our seven hybridomas and of the parental myeloma in pairwise comparisons in Figs. 4, 5, 6 and 7 and the resulting average clonal chromosome numbers in Table 1 and primary numbers in Tables 2 and 3:

**Table 2:** Chromosome copy numbers of five kayotypes (K) of mouse myeloma Ag8 and hybridomas Hyb cl-12, Hyb CN-13 and Hyb cl-9

Clone	Mouse Myeloma					Hybridoma CN-13					Hybridoma cl-12					Hybridoma cl-9				
Karyotypes	K1	K2	K3	K4	K5	K1	K2	K3	K4	K5	K1	K2	K3	K4	K5	K1	K2	K3	K4	K5
Chromosome Copy #	53	53	51	51	49	84	88	87	84	84	101	90	75	89	87	113	115	111	90	95
Chromosomes																				
1	1	1	2	1	1	3	3	3	3	3	3	3	3	3	3	4	4	4	3	4
2	1	1	1	1	1	3	3	3	3	3	4	3	2	3	2	4	4	4	3	4
3	0	0	0	0	0	1	1	1	1	1	3	2	2	2	2	2	2	1	2	2
4	1	1	1	1	1	2	2	3	3	2	3	3	1	4	3	2	3	3	3	3
5	1	1	1	1	1	3	3	3	3	3	3	3	1	3	2	4	4	4	3	3
6	0	0	0	0	0	2	2	2	1	2	2	2	1	2	1	3	3	3	2	2
7	0	0	0	0	0	2	2	2	2	2	4	2	2	2	2	2	2	2	2	2
8	1	1	1	1	1	4	3	3	3	4	4	3	2	3	3	3	4	4	3	3
9	1	1	1	1	1	3	3	3	3	3	3	3	2	3	3	5	5	5	3	3
10	1	1	1	1	1	3	3	3	2	3	3	3	3	3	3	4	4	2	3	3
11	2	2	2	2	2	3	3	3	3	3	6	4	3	4	4	5	5	5	4	4
12	0	0	0	0	0	1	2	1	1	1	2	2	2	2	1	2	2	2	2	2
13	1	1	1	1	1	3	3	3	3	3	4	3	3	3	3	4	3	4	3	3
14	2	2	2	2	1	2	3	3	3	2	4	4	4	4	4	5	6	8	5	5
15	2	1	1	2	2	4	3	4	4	4	4	4	4	4	4	6	6	5	4	4
16	1	1	1	1	1	3	3	3	3	3	4	4	3	3	3	3	3	3	3	3
17	1	1	1	1	1	3	3	3	3	3	3	3	2	3	3	3	3	3	3	3
18	0	0	0	0	0	1	1	1	1	1	4	2	2	3	2	2	2	2	2	2
19	2	2	2	2	2	3	3	3	3	3	4	4	3	3	4	4	6	4	4	4
X	0	0	0	0	0	2	2	2	2	2	2	2	1	2	2	2	2	2	2	2
Y	0	0	0	0	0	0	0	0	0	0	0	0	0	0	0	0	0	0	0	0
der(X;10)	1	1	1	1	1	1	1	1	1	1	1	1	1	1	1	2	2	2	1	2
der(2;X)	1	1	1	1	1	1	1	1	1	1	1	1	1	1	1	1	1	0	1	1
der(X;18)	1	1	1	1	1	1	1	1	1	1	1	1	1	0	1	1	1	1	1	1
der(19;X)	1	1	1	1	1	1	0	1	1	1	2	1	1	1	1	1	1	1	1	1
der(8;1)	1	1	1	1	0	0	1	1	1	0	1	1	1	1	1	0	0	0	1	1
der(13;1)	1	1	1	1	1	1	1	1	1	1	1	1	1	1	1	1	1	1	1	1
der(2;3;1)	1	1	1	1	1	1	1	1	1	1	1	1	1	1	1	2	2	2	1	1
der(1;3;7)	1	1	1	1	1	1	1	1	1	1	1	1	0	1	1	2	2	2	2	1
der(1;11)	1	1	1	1	1	1	1	1	1	1	1	1	1	1	1	2	1	2	1	1
der(17;1)	1	1	1	1	1	1	1	1	1	1	1	1	1	1	1	1	1	1	1	1
der(2;6)	1	1	1	1	1	0	1	0	1	0	1	1	1	1	1	1	1	1	0	0
der(2)	2	2	1	0	1	2	2	2	1	2	2	1	1	1	1	1	2	2	1	2
der(3;6)	1	1	1	1	1	1	1	1	1	1	1	1	1	1	1	2	2	2	1	1
der(3;4)	1	1	1	1	1	1	1	1	1	1	1	1	1	1	1	2	2	2	1	1
der(4;3)	1	1	1	1	1	1	1	1	1	1	0	0	0	0	0	1	1	1	1	1
der(4;5)	1	1	1	1	1	1	1	1	1	1	1	1	1	1	1	1	1	1	1	1
der(5;4)	1	1	1	1	1	1	1	1	1	1	1	1	1	1	0	1	1	1	1	1
der(12;5)	1	1	1	1	1	1	1	1	1	1	1	1	1	1	1	1	1	1	1	1
der(6;5)	1	1	1	1	1	0	1	1	1	0	1	1	1	1	1	1	1	0	0	1
der(17;6)	1	1	1	1	1	1	1	1	1	1	0	1	1	1	1	1	1	1	1	1
der(6;18;13)	1	1	1	1	1	1	1	1	1	1	1	1	1	1	1	1	1	1	1	1
der(11;6)	1	1	1	1	1	1	1	1	1	1	1	1	0	1	1	1	1	1	1	1
der(7)	1	1	1	1	1	1	1	1	1	1	0	1	1	1	1	1	1	1	1	1
der(8;14)	1	1	1	1	1	1	1	1	1	1	1	0	1	1	1	1	0	0	0	0
der(9;14)	1	1	1	1	1	1	1	1	1	1	1	1	1	1	1	1	1	1	1	1
der(12;10)	1	1	1	1	1	1	1	1	1	1	1	1	0	1	1	1	1	1	1	1
der(12;17)	2	2	1	2	1	3	3	3	3	3	2	2	2	2	2	3	2	3	2	2
der(14;8;1;13)	1	1	1	1	1	1	1	1	1	1	0	0	0	0	0	0	0	0	0	0
der(15)	2	2	2	2	2	2	4	2	2	2	2	2	2	2	2	3	3	3	2	3
der(16)	2	2	2	2	2	2	2	2	1	2	1	0	2	2	2	3	2	3	2	2
der(18;15)	1	1	1	1	1	1	1	1	1	1	0	1	1	0	1	1	1	1	1	1
Non-Clonal Markers	0	1	0	0	0	0	0	0	0	0	2	2	0	0	2	3	4	2	0	0

**Table 3:** Chromosome copy numbers of five karyotypes (K) of mouse hybridomas Hyb H12, Hyb F3, Hyb 94 and Hyb 1-5

Clone	Hybridoma H12					Hybridoma F3					Hybridoma 94					Hybridoma 1-5				
Karyotypes	K1	K2	K3	K4	K5	K1	K2	K3	K4	K5	K1	K2	K3	K4	K5	K1	K2	K3	K4	K5
Chromosome Copy #	71	70	71	68	75	77	78	80	81	81	71	77	71	71	82	107	105	93	89	99
Chromosomes																				
1	2	2	2	1	2	3	3	3	3	3	1	1	1	2	1	2	3	2	3	2
2	2	2	2	2	2	3	3	3	3	3	3	4	3	4	3	3	3	4	3	4
3	0	0	0	0	0	1	1	0	1	1	0	0	0	1	1	0	1	1	0	1
4	2	2	2	2	2	2	2	2	2	2	1	2	1	1	2	2	2	2	1	2
5	2	2	2	2	2	2	2	1	2	2	1	1	2	0	2	4	3	3	2	3
6	1	1	1	1	1	2	2	3	2	2	2	2	2	2	2	2	1	2	2	2
7	1	2	2	1	2	1	2	2	2	2	2	3	3	3	3	1	4	2	2	2
8	1	1	1	1	2	3	3	2	3	3	3	3	2	1	2	3	3	1	2	2
9	2	2	2	2	2	3	3	3	3	3	2	3	2	2	3	5	3	2	1	4
10	1	1	1	1	1	3	3	3	2	3	2	2	3	3	3	2	2	1	2	1
11	3	3	3	3	3	4	4	3	5	4	3	1	1	3	3	6	3	4	4	4
12	0	0	0	0	0	0	0	0	0	0	0	0	2	1	1	1	2	2	2	2
13	3	3	3	2	4	1	1	1	2	1	2	1	2	1	2	2	1	3	1	3
14	2	2	2	2	2	2	2	3	2	3	3	4	2	3	3	4	5	4	5	4
15	3	3	3	3	3	4	4	4	4	4	4	2	2	5	4	4	4	4	4	3
16	2	2	2	2	3	2	3	3	3	3	3	2	2	2	3	2	3	3	4	1
17	1	1	1	1	1	3	3	3	3	3	2	3	2	2	2	1	2	2	1	2
18	2	2	2	2	2	2	2	2	2	2	0	1	1	1	2	2	1	1	1	1
19	2	2	2	2	3	3	3	3	3	3	3	3	3	3	3	2	3	2	3	3
X	1	1	1	1	1	2	2	2	2	2	2	2	0	2	2	2	2	1	1	3
Y	0	0	0	0	0	0	0	0	0	0	0	0	0	0	0	0	0	0	0	0
der(X;10)	1	1	1	1	1	1	1	1	1	1	1	1	1	1	1	0	2	2	2	2
der(2;X)	1	1	1	1	1	0	0	0	0	0	0	0	0	0	0	1	1	2	2	1
der(X;18)	1	1	1	1	1	1	1	1	1	1	1	1	1	1	1	2	2	1	1	1
der(19;X)	1	1	0	1	1	1	1	1	1	1	1	1	1	1	1	1	2	2	1	2
der(8;1)	1	1	1	1	1	1	1	1	1	1	1	1	1	1	1	2	3	1	1	1
der(13;1)	1	1	1	1	0	1	1	1	1	1	2	2	1	1	1	1	1	1	0	1
der(2;3;1)	1	1	1	1	1	1	1	1	1	1	1	1	1	0	0	2	1	1	1	2
der(1;3;7)	1	1	1	1	1	1	1	1	1	1	2	1	1	0	1	1	2	2	1	2
der(1;11)	1	1	1	1	1	1	1	1	1	1	0	2	1	1	1	2	2	1	2	1
der(17;1)	1	1	1	1	0	1	1	1	0	1	1	0	0	1	1	2	2	2	0	2
der(2;6)	1	1	1	1	1	1	1	1	1	1	1	1	1	0	0	2	2	2	1	1
der(2)	2	2	2	2	0	1	2	1	1	1	2	2	2	3	0	4	0	1	2	2
der(3;6)	1	1	1	1	2	1	1	1	1	1	1	1	1	1	1	2	2	1	2	1
der(3;4)	1	1	1	1	1	1	1	1	1	1	1	1	1	1	1	2	2	2	2	2
der(4;3)	1	1	1	1	1	1	1	1	1	1	0	2	1	1	1	2	2	2	2	2
der(4;5)	1	1	1	1	1	1	1	1	1	1	1	1	1	1	0	0	0	0	0	0
der(5;4)	1	1	1	1	1	1	1	1	1	1	1	2	1	1	2	2	2	1	3	1
der(12;5)	1	1	1	1	1	1	1	1	1	1	1	1	0	1	1	1	2	1	1	1
der(6;5)	1	1	1	1	1	1	1	1	1	1	1	1	1	1	1	1	0	0	0	1
der(17;6)	1	1	1	1	1	0	1	1	2	1	1	1	1	0	1	2	2	2	1	2
der(6;18;13)	1	1	1	1	1	1	1	1	1	1	0	1	0	1	1	2	2	1	2	2
der(11;6)	1	1	1	1	1	1	1	1	1	1	1	1	1	1	1	2	2	2	2	2
der(7)	1	1	1	1	1	1	0	0	0	0	0	0	0	0	0	2	0	2	1	1
der(8;14)	1	0	1	1	1	1	1	1	1	1	0	1	0	1	1	1	0	0	1	1
der(9;14)	1	1	1	1	1	1	1	1	1	1	1	1	1	1	2	1	2	2	1	2
der(12;10)	1	1	1	1	1	1	1	1	0	1	0	1	0	1	1	2	1	2	1	2
der(12;17)	2	2	2	2	2	2	2	2	2	2	2	2	2	2	2	3	3	3	4	3
der(14;8;1;13)	1	0	1	1	1	1	1	1	0	1	0	0	0	0	0	2	1	1	1	1
der(15)	2	2	2	2	2	1	1	1	1	1	2	1	2	1	1	4	4	2	4	3
der(16)	2	2	2	2	2	2	0	2	3	3	2	2	2	1	2	4	4	1	1	1
der(18;15)	2	2	2	2	2	1	1	1	1	1	1	1	1	1	2	2	2	1	2	1
der(X;1)	0	0	0	1	1															
der(X;2;X;2?)	1	1	1	0	1															
mar(11;6)											1	1	1	1	1					
mar(1;11)											1	1	1	1	1					
Non-Clonal Markers	1	1	1	0	2	0	0	3	2	0	1	1	6	0	4	0	1	3	0	3

*Karyotype-arrays of myeloma Ag8 and hybridoma Hyb CN-13 ab +* (Fig. 4a, b). As can be seen in Fig. 4a and in Tables 1 and 2 most chromosomes of five karyotypes of myeloma Ag8 arrayed in panel (a) and of hybridoma CN-13 ab + arrayed in panel (b) formed parallel lines and are thus clonal. The resulting percentages of clonalities are listed on the x-axis of the arrays, above the respective chromosome numbers. With few exceptions they were predominantly 80 to 100% clonal. At the same time minorities of some chromosomes were non-clonal, differing from the majority of clonal counterparts mostly in the gains or losses of single chromosomes as shown in Fig. 4 and listed in Table 2.

Moreover the comparison of the two arrays shows the individualities of the two clones, and also their similarities. These similarities consisted primarily of 31 highly clonal and highly abnormal marker chromosomes shared by myeloma Ag8 and hybridoma CN-13 ab+. Further, the myeloma lacked several normal mouse chromosomes and shared all of its normal murine chromosomes with the hybridoma CN-13 ab+, although at lower copy numbers than in the hybridoma. The individualities and commonalities of the two karyotype-arrays thus confirmed the preliminary results of the single karyotypes of these clones shown above in Fig. 3a, b., namely that the myeloma had gained 33 new clonal chromosomes in its conversion to hybridoma CN-13 ab + (Table 1). The relatively high numerical gain of chromosomes by the hybridoma compared to the parental myeloma in the short times of fusion thus supports the single-step theory of progression.

*Karyotype-arrays of hybridomas Hyb cl-12 ab + and Hyb cl-9 ab +* (Fig. 5a, b)*.* As can be seen in Fig. 5 (and Table 2), the copy numbers of most chromosomes of the karyotypes of Hyb cl-12 ab + and of Hyb cl-9 ab + formed parallel lines and are thus quasi-clonal. The prevailing 60 to 100% clonalities of the chromosomes are listed on the x-axis of the arrays, above the respective chromosome numbers. At the same time the copy number of the remaining non-clonal minorities of certain chromosomes typically differed from the majority of clonal counterparts mostly in the gains or losses of single chromosomes as shown in Fig. 5 and in Table 2.

Moreover comparison of the two arrays shows the individualities of the two clones and also their similarities. These similarities consisted again primarily of the 31 highly clonal, myeloma-specific marker chromosomes, which are also shared with the hybridoma shown in Fig. 4. This is further correlative evidence that the 31 myeloma-specific marker chromosomes encode the common, myeloma-specific immortality [30]**.**

Further, the two hybridomas Hyb cl-12 ab + and Hyb cl-9 ab + shared with each other and with hybridoma CN-13 ab + all normal murine chromosomes, but mostly at hyper-diploid copy numbers. This suggests that probably more than one mouse B-cells were fused with the myeloma parent in the formation of these hybridomas.

With regard to the mechanism of progression, we emphasize again that the average clonal chromosome copy number of hybridoma cl-12ab + was 86 and that of hybridoma cl-9 ab + was 105. These hybridomas thus differ from the parental myeloma in 34 and 53 additional chromosomes respectively (Tables 1 and 2). These relatively high numerical gains of chromosomes by the hybridomas compared to the parental myeloma in the short times of fusions again support the single-step theory of progression.

*Karyotype-arrays of hybridomas Hyb H12 ab- and Hyb F3 ab-* (Fig. 6a, b)*.* As can be seen in Fig. 6, the copy numbers of most chromosomes of the karyotypes of hybridomas Hyb H12 ab- and Hyb F3 ab- formed parallel lines. The exact percentages of the clonalities of the chromosomes ranged between 60 to 100% as listed on the x-axis of the arrays above the respective chromosome numbers. The corresponding chromosomes are thus quasi-clonal. At the same time the copy number of non-clonal minorities of these chromosomes typically differed from the majority of clonal counterparts mostly in the gains or losses of single chromosomes, as shown in Fig. 6 and listed in Table 3.

Moreover comparison of the two arrays shows the individualities of the two clones and also their similarities. Again these similarities consisted primarily of the 31 highly clonal, myeloma-specific marker chromosomes, which are also shared with the three hybridomas shown in Figs. 4 and 5 (and those shown in Fig. 7 below). This confirms again the view that the 31 myeloma-specific marker chromosomes encode the common, myeloma-specific neoplastic immortality [30]. Further, the two antibody-negative (ab-) hybridomas H12 ab- and F3 ab- both lacked mouse chromosome 12. Notably chromosome 12 is also missing in the parental myeloma (Fig. 4a) and is known to encode the heavy chain of moues antibodies [36, 39, 40]. In view of this, we pointed out above that the absence of intact chromosome 12 in Hyb H12 ab- and Hyb F3 ab- and the lack of functional antibody in the parental myeloma Ag8 indicate that these two hybridomas must both be antibody-negative (see Results, Clonal phenotypes of hybridoma). As expected, the individual and common chromosomes of Hyb H12 ab- and Hyb F3 ab- shown above in the karyotypes of Fig. 3c, d. confirmed and extended the patterns of the two arrays shown here, namely that hybridomas contained numerous new chromosomes compared to the parental myeloma.

With regard to the mechanism of progression, we emphasize again that the numbers of clonal chromosomes of the hybridoma H12 ab- are 71 and those of F3 ab- are 79 (Tables 1 and 3) and are thus significantly higher than the 52 chromosomes of the parental myeloma Ag8. They differed from the parental myeloma in 19 and 27 additional, clonal chromosomes (Tables 1 and 3). These relatively high numerical gains of chromosomes by the hybridomas compared to the parental myeloma in the short times of fusions thus support again the single-step theory of progression.

*Karyotype-arrays of hybridomas Hyb 94 and Hyb 1-5 (*Fig. 7a, b*)***.** As can be seen in Fig. 7a (and Table 3) the individual chromosome numbers of the five hybridoma Hyb 94 cells analyzed formed several non-parallel lines and accordingly ranged from 71 to 82 chromosomes per cell - for a clonal average of 74 (Table 1). This hybridoma is thus clonally heterogeneous. Nevertheless, all five Hyb 94 karyotypes shared two Hyb 94-specific marker chromosomes and all but three of the 31 myeloma-specific chromosomes (Table 3). The Hyb 94 karyotypes are thus quasi-clonal, with copy numbers ranging from 40 to 100% clonality (Fig. 7a). The simplest explanation for the relatively high clonal heterogeneity of Hyb 94 suggests that this clone is a sub-clonal precursor of a hybridoma that is losing non-clonogenic chromosomes after it originated from a fusion of myeloma with B-cells. Such clonal heterogeneity has also been observed previously in metastases of solid cancers [11].

As shown in Fig. 7b, the karyotype array of Hyb 1-5 was also relatively heterogeneous. The clonality of chromosome numbers ranged from 40 to 100% and averaged at about 60% (Fig. 7b). Nevertheless, all five Hyb 1-5 karyotypes shared all but one of the 31 myeloma-specific chromosomes (Table 3). The simplest explanation for the high clonal heterogeneity of Hyb 1-5 suggests again that this clone, like Hyb 94 above, is a heterogeneous precursor of a prospective hybridoma that is losing non-clonogenic chromosomes after it originated from an unstable fusion of myeloma with B-cells.

With regard to the mechanism of progression, we emphasize again that the average numbers of quasi-clonal chromosomes of hybridoma Hyb 94, namely 74, and of Hyb 1-5, namely 99, differed from the parental set of myeloma chromosomes by 22 and 47 additional chromosomes respectively (Table 1). This multiplicity of newly acquired chromosomes during the short fusion events again supports a single step model of fusion-mediated neoplastic progression, which continued to evolve after fusion.

## Discussion

Multiple studies including ours have found “late” but also “immediate” progressions of cancers with numerous new, progression-specific chromosomes [14, 25, 41]. However no intermediates or prospective progressions with subsets of new progression-specific chromosomes were reported. In view of this and the existence of “immediate” progressions with numerous new chromosomes we have advanced here the theory that neoplastic progressions are saltational events, in which all chromosomes of progressions are united in single steps. To test this saltational theory, we asked here, whether the numerous new chromosomes of most neoplastic progressions are acquired gradually or simultaneously in single steps.

### Simultaneous acquisitions of numerous new chromosomes convert myelomas to immortal hybridomas in single steps

In view of evidence that neoplastic progressions of certain cancers, notably immortal hybridomas from myelomas can be generated within a few minutes by fusions of heterologous cells, we tested our saltational theory by analyses of the chromosomes of seven hybridomas for new hybridoma-specific chromosomes and for the absence of detectable intermediates.

As shown in Table 1, our experiments demonstrated that seven individual and immortal hybridomas had indeed gained from 19 to 53 chromosomes from fusions with B-cells within a few minutes – and that there were no detectable intermediates. We also show in Table 1 that these seven hybridomas differed from a theoretical parental hybrid of 92 chromosomes (52 myeloma and 40 B-cell chromosomes) in gains of 13 to losses of 21 chromosomes. These discrepancies between the experimental and theoretical sums of chromosome numbers confirmed original observations of Kohler and Milstein and subsequent studies by Wollweber et al. [23, 26].

In view of these results, we conclude that hybridomas are generated by haphazard combinations of the chromosomes of fused cells in single steps. This conclusion explains the fast kinetics of hybridomagenesis, the absence of karyotypic intermediates, the low yields of only about one viable hybridoma per 10^4-5 fused cells (Background), and the individuality of the resulting hybridomas described here and previously (Background and references [9, 11, 23, 26, 30]).

### Are saltational single-step mechanisms also generating spontaneous, late neoplastic progressions?

The following rare observations on the origin of spontaneous neoplastic progressions also support the saltational theory of neoplastic progressions:

1. Distinguishing between paternal and maternal chromosomes by restriction length polymorphisms Onodera et al. found in 1992 highly symmetric distributions of paternal and maternal chromosomes in hyperdiploid leukemias. The authors concluded that, “These results suggest that the hyperdiploid karyotype usually arises by simultaneous gain of chromosomes from a diploid karyotype during a single abnormal cell division” [42]. This study was confirmed and extended by Paulsson et al. in 2005 [43].
2. Studying progression of prostate cancers in 2013 Baca et al. detected “considerable genomic derangement over relatively few events in prostate cancer and other neoplasms, supporting a model of punctuated cancer evolution.” [44].
3. Stepanenko et al. observed in 2015 that, “Transfection of either the empty vector pcDNA3.1 or pcDNA3.1 CHI3L1 (a growth factor) into 293- cells (a human embryo kidney cell line) initiated the punctuated genome changes” of simultaneous gains and losses of chromosomes [12].
4. Studying the progression of breast cancers Gao et al. observed by whole genome sequencing in 2016, “Despite profiling hundreds of single cells from many spatial regions, we did not detect any intermediate copy number profiles, indicative of gradual evolution,” and concluded, “our data challenge the paradigm of gradual evolution” [45].
5. In a comparison of single with multi-hit or “linear” theories of metastatic progressions in 2016 Turajlic and Swanton conclude, “It is conceivable that macroevolutionary leaps (large-scale genomic alterations) could catalyze all the steps to metastases, especially in narrow time frames” [46] – much as those studied by us here.

Further we have shown previously that spontaneous metastatic and drug-resistant progressions have individual clonal karyotypes with numerous progression-specific chomosomes [11, 34], just as the hybridomas studied here. The individuality, complexity and clonality of the karyotypes [9–11] and transcriptomes [10, 32] of spontneous progressions indicate, however, a saltational, speciation-type of event [9, 47, 48] – much like the saltational events we found here for hybridomas.

It would appear then that saltational, single step mechanisms could generate rare progressions “early” and “late” by spontaneous karyotypic rearrangements (see Background, Alternative single-step theory of progression), independent of cell fusions. Accordingly the typically long latencies between cancers and progressions would simply reflect the low probabilities of speciation by random karyotypic variations.

Nevertheless, there is also sporadic evidence for a role of cell fusions in spontaneous progressions based on several independent studies that were recently reviewed by Lazebnick [49].

Finally, it did not escape our attention that the single-step theory of progression or subspeciation of cancers advanced here and previously [7, 10, 11, 33, 34, 48] derives independent support from chromosomal theories postulating that conventional speciations or subspeciations also occur in single saltational steps - without stable intermediates [47, 50–53].

## Conclusions

We conclude that the evidence from the hybridoma model tested here and the independent observations of others including us about spontaneous clonal progressions are based on saltational recombinations of cancer chromosomes or of cancer chromosomes with chromosomes of heterologous cells. This model encourages the following clinically relevant conclusions:

1. Our analysis of the karyotypic basis of progressions here and previously [9–11] indicates that the progressions of cancers are clonal and thus probably the proximate causes of neoplastic progressions. This conclusion confirms and extends a prior prediction of Heng et al. [54].
2. The inherent karyotypic variability of cancer- and progression-specific aneuploidy (Background) thus explains and supports Foulds’ rule, that “progression does not always reach an end-point within the life-span of the host” [1], and Nowell’s similar observation, “that the process is a continuing one” [41]. Therefore, we conclude that progressions of progressions are a lasting concern [1, 12], particularly since progressions are responsible for 90% of the mortality of cancers [55, 56].
3. Further we propose that the degree of cancer-specific aneuploidy predicts the clinical risk of neoplastic progression, because cancer-specific aneuploidy catalyzes karyotypic variation. This view thus confirms and extends classical predictions based on DNA content [57–61].

## Methods

### Preparation of hybridomas

Thymidine-kinase deficient myeloma Ag8 cells and B-cells from mice, induced to produce antibodies with specific antigens, were fused at equal numbers for about 5-10 min in about 50% polyethylene glycol following established methods of Zeitler and Beatty (UC Berkeley, above) and of the literature [26, 35, 36]. After fusions the cells were washed and incubated at 37 C for one to 2 days in selective medium containing aminopterin, which inhibits natural thymidine synthesis and thymidine, which substitutes lacking thymidine after fusion with B-cells (Sigma Co, St Louis, MO or ATCC, Rockville, MD). In these conditions fused Ag8 myeloma cells survive from added thymidine picked up by B-cell-derived thymidine kinase. Within a few days after fusion, un-fused myeloma cells die out due to lack of thymidine and toxicity of aminopterin, and un-fused B-cells perish spontaneously within several generations in culture. At that time normal medium was used for the propagation of surviving hybridoma cells. One to 2 weeks later microscopic clones appeared, which were then sub-cultured in conventional RPMI 1640 medium supplemented with 10% fetal calf serum following published procedures [23, 26, 35, 36]. Clonal cultures of immortalized myeloma-B-cell hybrids arose from fusions at rates of about one hybridoma per 10^4 to 5 myeloma cells. Hybridoma cells were then propagated in suspension cultures in RPMI 1640 medium supplemented with 10% to 20% fetal calf serum and 1% of 100× *Antibiotic Antimycotic* (Sigma Co, St Louis, USA).

### Karyotypic analyses myeloma and hybridoma cells

One to 2 days before karyotyping, cells were seeded at about 50% confluence in a 5-cm culture dish with 3 ml of the medium described above. After reaching ~75% quasi-confluence, 250–300 ng colcemid in 25–30 μl solution (KaryoMax, Gibco) was added to 3 ml medium. The culture was then incubated at 37 °C for 4–8 h. Subsequently cells were washed twice with 3 ml of physiological saline and then incubated in 0.075 M KCl at 37 °C for 15 min. The cell suspension was then cooled in ice-water, mixed (‘prefixed’) with 0.1 volume of the freshly mixed glacial acetic acid-methanol (1:3, vol. per vol.) and centrifuged at 800 g for 6 min at room temperature. The cell pellet was then suspended in about 100 μl supernatant and mixed drop-wise with 5 ml of the ice-cold acetic acid-methanol solution and then incubated at room temperature for 15–30 min or overnight at 5C. This cell suspension was then pelleted and was then either once more re-suspended in fixative and pelleted, or was directly re-suspended in a small volume of the acetic acid-methanol solution for microscopic examination. For this purpose an aliquot of a visually turbid suspension was transferred with a micropipette tip to a glass microscope slide, allowed to evaporate at room temperature and inspected under the microscope at 200× for an adequate, non-overlapping density of metaphase chromosomes. Metaphase chromosomes attached to glass slides were then hybridized to color-coded, mouse chromosome-specific DNA probes as described by the manufacturer, *MetaSystems* (Newton, MA 02458). Chromosomes were then sorted into conventional karyotypes with a computerized Zeiss Imager M1 microscope, programmed by *MetaSystems* (Newton, MA 02458) following published procedures [11, 33, 34, 61].
